# Improvement of BMI after Lifestyle Intervention Is Associated with Normalisation of Elevated ELF Score and Liver Stiffness in Obese Children

**DOI:** 10.1155/2015/457473

**Published:** 2015-07-26

**Authors:** Imeke Goldschmidt, André Di Nanni, Carolin Streckenbach, Kerstin Schnell, Thomas Danne, Ulrich Baumann

**Affiliations:** ^1^Paediatric Gastroenterology and Hepatology, Children's Hospital, Hannover Medical School, 39625 Hannover, Germany; ^2^Children's Hospital “Auf der Bult”, 30173 Hannover, Germany

## Abstract

*Background*. Noninvasive tools to diagnose nonalcoholic fatty liver disease (NAFLD), including transient elastography (TE) and enhanced liver fibrosis panel (ELF), have only been evaluated in children with biopsy-proven NAFLD. We evaluated the prevalence of ELF and TE abnormalities in obese children without clinical liver disease and examined the effects of BMI stabilization on ELF and TE in a longitudinal approach. *Methods*. 39 obese children (17 m, age 12.3 (7.6–17.4) years) who participated in a 12-month lifestyle-intervention program underwent TE and ELF testing at baseline and at completion of the program. Results were compared with data from a nonobese paediatric cohort. *Results*. TE and ELF at baseline were significantly elevated compared to controls (TE: 5.9 (3.4–8.3) kPa versus 4.45 (2.45–8.85) kPa, *P* < 0.01; ELF: 9.0 (7.87–9.60) versus 8.6 (7.33–11.52), *P* = 0.033). All children with elevated TE and ELF results had normal transaminases. After the program, ELF and TE normalized. Reduction of ELF and TE was associated with a decrease in BMI centile. *Conclusion*. Abnormal TE and ELF results in obese children suggest presence of NAFLD even when transaminases are normal. TE and ELF might be used as monitoring tools for NAFLD. BMI stabilisation normalizes TE and ELF, underlining the impact of lifestyle intervention.

## 1. Introduction

Childhood obesity represents a growing problem in industrialized nations. It can be accompanied by a number of comorbidities, including metabolic syndrome with arterial hypertension, type 2 diabetes, dyslipidemia, and nonalcoholic fatty liver disease (NAFLD) or steatohepatitis (NASH) [1]. NASH/NAFLD has now become the most frequent cause for chronic liver disease in children in industrialised nations [2]. According to some reports, NAFLD appears to be prevalent in up to 80% of obese children [2, 3] and can progress to hepatic cirrhosis requiring liver transplantation in individual cases [4].

Elevated transaminases are a frequent feature in obese children (up to 10% in children with BMI ≥ 95th centile) [5]. The clinical challenge lies in determining who should undergo more detailed investigations for NAFLD and NASH, which in suspicion of advanced disease still comprise liver biopsy [6].

Several noninvasive approaches in obese children have been evaluated in order to screen for NAFLD and NASH [7–15]. The majority of these studies have been conducted in children with an established diagnosis of obesity-related liver disease based on liver biopsy. The prevalence of abnormal test results for noninvasive tests in obese children with no other indication of liver disease is unclear. A screening tool that would permit identification of those children at risk of obesity-related liver disease would facilitate the streamlining of diagnostic procedures and the selection of candidates for more invasive diagnostics and for continued surveillance and might also serve as an additional motivator for the implementation of life-style changes.

Our study examines the use of two noninvasive techniques in obese children, namely, liver elastography by Fibroscan and serological determination of the enhanced liver fibrosis (ELF) score, in a longitudinal approach. We examined the prevalence of abnormal test results for ELF and Fibroscan in an unselected cohort of obese children. Furthermore, we have studied the development of these markers after a structured nutrition-exercise-behaviour-intervention program for obese children in order to see whether improvement of markers of obesity will result in improvement in noninvasive markers of associated liver disease.

## 2. Subjects and Methods

### 2.1. Patients

Between February 2011 and August 2012, all children who had been enrolled in the lifestyle-intervention program “KiCK” at Children's Hospital “Auf der Bult” in Hannover targeting childhood obesity were approached for participation in our study. Of 48 eligible children, 39 (age 7.6–17.4 years, median 12.3 years, 17 boys, 22 girls) agreed to participate. Demographic and baseline clinical characteristics of the participants are described in Table 1.

Study participants underwent Fibroscan examination and blood sampling for ELF testing on the occasion of routine visits at the beginning and at the end of the 12-month lifestyle-intervention program. Medical background data and routine laboratory results were extracted from the patients' notes.

### 2.2. Controls

*N* = 85 healthy children (33 girls and 52 boys, age 3.8 (0.3–15.8) years) who underwent blood sampling at the occasion of minor surgery were recruited as normal controls for ELF test. Normal values for transient elastography (TE) were drawn from a cohort of 270 healthy children (153 boys, 117 girls) aged 0.3–17.7 (median 7.0) years as published by our group in [16].

Informed consent was obtained from the patients' and controls' legal guardians and the patients/controls themselves if appropriate.

The study protocol was approved by the local ethics board of Hannover Medical School and is in compliance with the Helsinki Declaration of the World Medical Association.

### 2.3. Lifestyle-Intervention Program

The lifestyle-intervention program “KiCK” targets obese children between the ages of 8 and 17 years. It comprises a thorough medical examination, a psychological evaluation, weekly group meetings with exercise sessions, and regular educational activities covering topics such as healthy eating, nutrition behavior, and dealing with everyday problems. Parents and guardians are included in the educational activities. The program runs for a total of 12 months. Participants are subsequently followed up in a dedicated obesity clinic. Only children with a BMI > 97th centile are eligible for participation.

### 2.4. Liver Stiffness Measurements

Liver stiffness measurements (LSM) were performed by transient elastography (TE) using the Fibroscan (Echosens, Paris, France). Echosens provides two measurement probes (S, M), which will perform elastography in varying depths ranging from 15–40 mm (S1) and 20–50 mm (S2) to 25–65 mm (M). For the vast majority of patients, the M probe was used based on the manufacturer's recommendation for probe choice according to chest circumference (CC) and age (CC < 45 cm or age < 6 years S1 probe, CC 45–75 cm or age 6–14 years S2 probe, and CC > 75 cm or age > 14 years M probe). An XL probe was not available at the time of the study.

For the liver stiffness measurements, patients were in supine position with the right arm in abduction. Manufacturer recommendation is to perform Fibroscan measurements in the anterior axillary line. Depending on distribution of subcutaneous fat in this particular study population, we sometimes had to deviate from this and perform measurements in the mid-clavicular line.

Fibroscan results were only considered acceptable if the following conditions were met: at least 8 valid measurements were obtained in one particular site of measurement (met in 30 patients at the baseline visit and in 21 at the follow-up visit) and the ratio of the median of ten successive measurements and the interquartile range (IQR) was <30% (failed in 2 cases with valid number of measurements). A total of 49 acceptable Fibroscan examinations were obtained in *n* = 39 patients (28 at baseline, 21 at follow-up).

### 2.5. Enhanced Liver Fibrosis (ELF) Test

The enhanced liver fibrosis test (ELF test, Siemens) combines hyaluronic acid (HA), amino-terminal propeptide of type III collagen (PIIINP), and tissue inhibitor of metalloproteinase-1 (TIMP-1) in an algorithm. According to the manufacturers, the following cut-off values are applicable: values <7.7 for mild or no fibrosis, 7.7–9.8 for moderate fibrosis, and >9.8 for severe fibrosis. Serum samples for ELF testing were obtained in *n* = 33 patients for the baseline visit and in 27 patients for the follow-up visit. Samples were frozen and stored at −20°C until time of analysis. Siemens ADVIA Centaur was used for analysis.

### 2.6. Statistics

Continuous variables are given as median and range. For categorical variables, frequencies and percentages are given. Wilcoxon signed-rank test was used to compare paired data from before and after the lifestyle-intervention program. Chi-square test was used to compare frequencies of categorical variables in different groups.

## 3. Results

### 3.1. Degree of Obesity and Associated Medical Issues at Baseline

39 children were available for baseline assessment before the start of the KiCK program. Baseline parameters of obesity are described in Table 1. 34/39 children had a BMI above the required 97th centile at baseline.

We determined homeostasis model assessment (HOMA) index as a marker for the presence of insulin resistance and fasting triglycerides and cholesterol as a marker for dyslipidemia. HOMA index was found to be above normal (i.e., >1) in the whole study population and in a range suggestive of insulin resistance (>3) in *n* = 21. Dyslipidemia with raised triglyceride and/or cholesterol levels was present in 9 and 5 children, respectively; 16 and 12 children, respectively, had raised LDL cholesterol. Based on these findings, 9 children fulfilled the definition of having metabolic syndrome, as defined by a cooccurrence of three or more features of obesity with waist circumference (WC) > 90th centile, dyslipidemia, alterations of glucose metabolism, and hypertension.

### 3.2. Hepatic Involvement at Baseline

Liver function tests and results of TE and ELF test are given in Table 2. ALT, AST, and GGT were normal in the majority of children, giving no indication of obesity-related liver disease. However, liver stiffness values were found to be significantly elevated compared to healthy controls (5.9 (3.4–8.3) kPa versus 4.45 (2.45–8.85) kPa, *P* < 0.01). 28.6% (*n* = 8) of our study participants showed liver stiffness values above the upper limit of normal (6.5 kPa).

ELF values were equally found to be significantly higher in the obese study participants when compared to healthy controls. 21.2% (*n* = 7) of our participants had ELF values above the cut-off of 9.28 given as the cut-off for “any fibrosis” in [13].

### 3.3. Change in Anthropometric Measures and Laboratory Values after 12 Months of Lifestyle-Intervention Program

**Figure 1:**
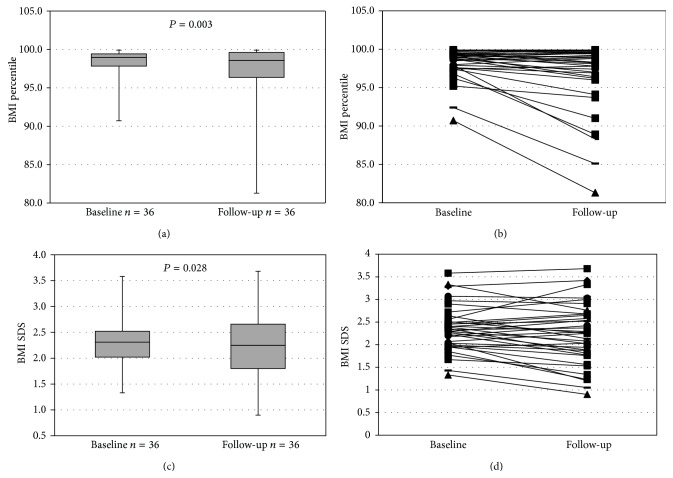
BMI at baseline and follow-up.

Pre- and postintervention data were available for 36 children. After 12 months of lifestyle intervention, an overall significant improvement for BMI percentile and BMI-SDS could be observed (Table 3), even though individual patients failed the program completely (Figure 1). There was a nonsignificant trend for HOMA index to improve (*P* = 0.09). While triglyceride levels improved significantly (*P* = 0.02), neither total cholesterol nor LDL cholesterol showed a significant change. ALT levels stayed in the range of normal for the majority of patients but nevertheless showed a significant increase (*P* = 0.02).

### 3.4. Change in Hepatic Parameters after 12 Months of Lifestyle-Intervention Program

**Figure 2:**
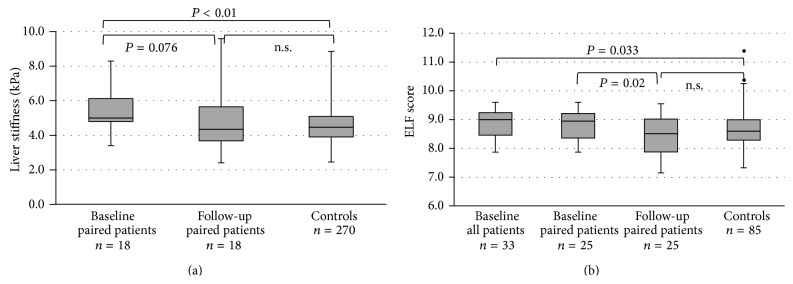
ELF and TE at baseline and follow-up.

Liver stiffness values improved after the program, although this difference failed to reach the level of significance (*P* = 0.076). Postprogram liver stiffness values did not differ from control values any more (Figure 2(a)). ELF values improved significantly (*P* = 0.02) and were similarly not different from control values any more (Figure 2(b)).

### 3.5. Influences on Improvement of ELF Scores and Liver Stiffness Measurements

**Figure 3:**
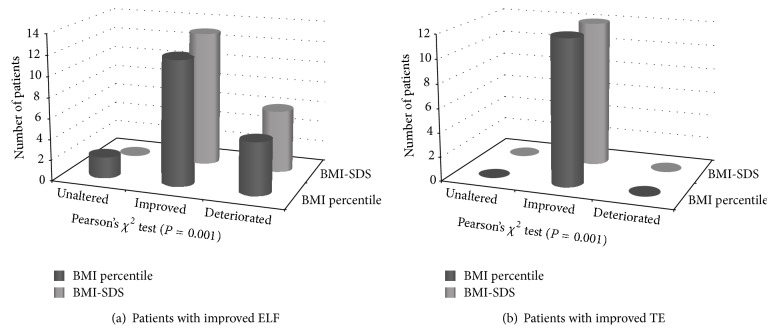
Changes in BMI according to improvement in TE and ELF.

Improvement of BMI centiles, BMI-SDS, and WHtR was significantly associated with a decrease in liver stiffness (Table 4, *P* = 0.001, chi-square). Similarly, a decrease in BMI centiles and BMI-SDS was significantly associated with a decrease in ELF test (Figures 3(a) and 3(b)). Decrease in HOMA index did not show any association with ELF or TE improvement.

## 4. Discussion

In our study, we addressed the issue of whether the noninvasive markers transient elastography as measured by Fibroscan and enhanced liver fibrosis panel ELF could serve as screening tools for hepatic involvement in obese children who otherwise show no indication of liver disease. We also wanted to examine the effects of potential BMI improvement on liver stiffness and ELF score, with a view that has effects on noninvasive parameters which might help to underline the overall health benefit of weight loss for these patients. To the best of our knowledge, this is the first study that looks at the development of ELF and TE in a longitudinal approach during weight loss.

NASH and NAFLD can be difficult to diagnose in obese children and adolescents. Usually, elevated transaminases are used as a screening tool for NAFLD [17, 18]. According to current ESPGHAN recommendations, a diagnosis is then made based on ultrasound findings, age of the child, concurrent risk factors, exclusion of other liver diseases, and possibly liver biopsy. About 10% of children are with BMI ≥ 95th centile present with elevated transaminases [5]. However, NAFLD has also been found in patients with normal transaminases [2, 19]. A screening based solely on levels of transaminases therefore risks missing children who have or are at risk of developing NAFLD. Noninvasive screening tools could help to identify children that merit further surveillance and investigation.

We found both liver stiffness and ELF score to be significantly elevated in obese children compared to a healthy nonobese control sample. Almost one-third of obese children had increased liver stiffness values initially, and 20% had ELF values that have previously been described as indicative of hepatic fibrosis [10, 13]. None of the children with abnormal TE or ELF test results had abnormal transaminases, meaning that ELF and TE were the only parameters indicating liver affection.

Maximal TE values were 8.3 kPa. The cut-off for the diagnosis of significant fibrosis in an adolescent NAFLD population has been previously reported at 7.4 kPa [12] and 8.6 kPA [9], while values between 5 and 7 kPa suggested presence of abnormality [12]. The drawback is that transient elastography, while good at detecting advanced fibrosis with high sensitivity, lacks the power to precisely discriminate low degrees of fibrosis. It is therefore impossible to precisely determine the exact severity of hepatic changes in the children of our cohort. Based on the publications by Nobili and Alkhouri [9, 12], however, we have to assume that the elevated TE results reflect a certain degree of hepatic steatosis and/or fibrosis that would otherwise have been undetected. A comparison of our liver stiffness results for histology of course would have been ideal but was impossible for ethical reasons.

As for ELF, normal ranges for children and adolescents have not been published previously. Normal values in adults were found to be dependent on age and gender [20], with higher values in men and in adults >30 years versus <30 years of age. Our own control cohort is too small to calculate centiles according to age and gender. Based on the findings of Lichtinghagen et al. [20], it is conceivable that the postulated difference between ELF values in the obese cohort versus the control cohort results in part from an age difference between the groups. However, the age of the study population in [13] giving a cut-off of 9.28 for the presence of any fibrosis in adolescents with biopsy-proven NASH/NAFLD was very similar to the age of our cohort.

As for any test, its diagnostic value and accuracy depend on the characteristics of the patient sample and the clinical question it is used for. Other studies that evaluated the use of TE or ELF for the diagnosis of NASH/NAFLD used preselected patient cohorts that had biopsy-proven NAFLD [7–15]. While cut-offs and their interpretation from these diagnostic studies cannot be unquestioningly transferred to screening populations like our cohort, our findings still show that there can be hepatic effect in obesity that might otherwise go unrecognized.

The current ESPGHAN guidelines for the diagnosis of NAFLD/NASH describe the following indications for liver biopsy: to rule out other treatable diseases, in case of clinically suspected advanced liver disease, before pharmacological or surgical treatment and as part of structured intervention protocols or clinical trials [14]. ELF and TE could help to identify patients for heightened NASH/NAFLD surveillance and possible liver biopsy.

After the program, only 2 children still had liver stiffness values above the upper limit of normal, and ELF decreased significantly. One limitation here is certainly the small amount of paired data due to technical failure of the Fibroscan examination in some of the patients. The reduced success rate of TE in obese patients has been reported repeatedly [16, 21]. The use of an XL probe would have been ideal; however, this probe was not available yet at the time of the study. Also, differences in liver stiffness results, according to which probe is used, have been described [16, 22]. We therefore consider it an advantage that both the obese cohort and the control children were examined with the M probe. The limited number of paired pre- and post-TE exams might explain why the pre-/postdifference failed to reach the level of significance.

Another limitation of this study is the low overall impact of the structured lifestyle-intervention program. While there was a clear-cut effect on BMI centile, this effect in total was small and many children stayed well in the obese spectrum. This success rate is comparable to other published reports on the feasibility and effects of lifestyle-intervention programs [23–26]. The KiCK program has previously been shown to be associated with an improvement in quality of life of the participants, which in part was independent of the extent of BMI reduction [27]. Despite the moderate BMI-stabilizing effect in our current series, most of the metabolic parameters either improved significantly or at least showed a trend towards improvement. Data on non-HDL cholesterol, which could have served as an additional metabolic predictor of hepatic injury [28], unfortunately was not available. A larger weight loss effect might have helped to establish a link between improvement of ELF and TE and weight loss parameters even more clearly. In adults, several lifestyle-intervention programs linked weight loss to an improvement in histological inflammation and partially in fibrosis [29]. In children, several trials have shown improvement of biochemical parameters such as ALT after BMI stabilisation, but only one trial exists that examined the effects of weight loss on histological changes [26]. The average weight loss seen in this trial was bigger than what we observed in our cohort. We demonstrated elevated markers of hepatic fibrosis in obese children even in the absence of elevated transaminases, with a normalisation of the mentioned markers after moderate BMI stabilisation. The changes in TE and ELF observed in our cohort might reflect true histological improvement. Our findings suggest that the success and influence of therapeutic intervention on obesity-related comorbidities could potentially be monitored noninvasively. The fact that we found a normalization of ELF and TE despite the small effects on BMI supports the statement that every little step of weight loss improves hepatic health, even if complete BMI normalization cannot be reached. This statement could potentially be used to support patient motivation.

## 5. Conclusion

ELF and TE can detect hepatic changes suggestive of NAFLD in obese children even when transaminases are normal. ELF and TE could therefore help to select children that warrant further surveillance, even after the end of a lifestyle-intervention program, and might need to undergo more invasive diagnostic measures in the future. A structured lifestyle-intervention program led to normalisation of liver stiffness and ELF results in association with a reduction in BMI centile and BMI-SDS. ELF and TE could therefore serve as monitoring tools for therapeutic intervention in children and adolescents with NAFLD or NASH.

## Figures and Tables

**Table 1:** Demographic and baseline clinical characteristics of the study population.

	*n* (%)/median (range)
Obese study population	
Gender	
Boys	17 (44%)
Girls	22 (56%)
Age (years)	12.3 (7.6–17.4)
Characteristics of obesity	
BMI (kg/m^*2*^)	28.48 (20.9–42.7)
BMI percentile	99.1 (90.7–99.9)
BMI-SDS	2.36 (1.33–3.58)
WHtR	0.61 (0.51–0.74)
WC > 90th centile	39 (100%)
Triglycerides (mg/dL)	80 (25–317)
Above ULN	9 (23.07%)
Total cholesterol (mg/dL)	177 (108–289)
Above ULN	5 (12.82%)
LDL cholesterol (mg/dL)	110 (44–225)
Above ULN	16 (41%)
Fasting glucose (mg/dL)	84 (69–107)
Above ULN	0 (0%)
HOMA index (*n* = 37)	3.2 (1.1–7.4)
>1	37 (100%)
>3	22 (59%)

BMI: body mass index; SDS: standard deviation score; WHtR: waist-to-height ratio; WC: waist circumference; ULN: upper limit of normal; HOMA: homeostasis model assessment; n.r.: not recorded.

Upper limits of normal: triglycerides 120** **mg/dL, cholesterin 250** **mg/dL, fasting glucose 120** **mg/dL, HOMA index <1, and LDL cholesterol 116** **mg/dL.

**Table 2:** Hepatic involvement in obese study group at baseline in comparison to healthy controls or laboratory reference range.

	Obese cohort median (range)	Healthy controls median (range)	*P*
ALT (ULN 39 U/L)	19 U/L (10–60)		

AST (ULN 41 U/L)	23 U/L (10–41)		

GGT (UNL 44 U/L)	22 U/L (3–44)		

Bilirubin (ULN 9 *μ*mol/L)	5 *μ*mol/L (2–14)		

Transient elastography (ULN 6.5 kPa)	5.9 kPa (3.40–8.30) *n* = 28 Above ULN: *n* = 8 (28%)	4.45 kPa (2.45–8.85) *n* = 270	<0.01
Enhanced liver fibrosis (ELF) test cut-off for “any” fibrosis: 9.28	9.0 (7.87–9.60) *n* = 33 Above 9.28: *n* = 7 (21%)	8.6 (7.33–11.52) *N* = 85	0.033

ALT: alanine aminotransferase; AST: aspartate aminotransferase; GGT: gamma-glutaryl transpeptidase; ULN: upper limit of normal; TE: transient elastography; ELF: enhanced liver fibrosis panel.

**Table 3:** Anthropometric measures and laboratory values at baseline and after 12 months.

	*n*	Obese study population, baseline, median (range)	*n*	Obese study population, follow-up, median (range)	*P*
BMI					
All participants	39	28.48 (20.9–42.7)			
Patients with paired follow-up data	36	28.13 (20.9–42.7)	36	27.79 (19.2–44.9)	0.925
BMI percentile					
All participants	39	99.1 (90.7–99.9)			
Patients with paired follow-up data	36	98.95 (90.7–99.9)	36	98.55 (81.3–99.9)	0.003
BMI-SDS					
All participants	39	2.36 (1.33–3.58)			
Patients with paired follow-up data	36	2.31 (1.33–3.58)	36	2.25 (0.90–3.68)	0.028
WHtR					
All participants	39	0.61 (0.51–0.74)			
Patients with paired follow-up data	33	0.60 (0.51–0.73)	33	0.60 (0.47–0.77)	0.914
Triglyceride					
All participants	39	80 mg/dL (25–317)			
Patients with paired follow-up data	36	81 mg/dL (25–317)	36	61 mg/dL (22–328)	0.020
Total cholesterol					
All participants	39	177 mg/dL (108–289)			
Patients with paired follow-up data	36	180 mg/dL (108–289)	36	166 mg/dL (108–265)	0.090
LDL cholesterol					
All participants	39	110 mg/dL (44–225)			
Patients with paired follow-up data	36	110 mg/dL (44–225)	36	104 mg/dL (48–207)	
Fasting glucose					
All participants	39	84 mg/dL (69–107)			
Patients with paired follow-up data	36	84 mg/dL (69–107)	36	81 mg/dL (67–97)	0.002
HOMA index					
All participants	37	3.2 (1.1–7.4)	31	2.4 (0.3–7.1)	
Patients with paired follow-up data	29	3.2 (1.1–7.4)	29	2.3 (0.3–7.1)	0.091
Transient elastography					
All participants	28	5.9 kPa (3.4–8.3)	21	4.5 kPa (2.4–9.6)	
Patients with paired follow-up data	18	5.0 kPa (3.4–8.3)	18	4.4 kPa (2.4–9.6)	0.076
Enhanced liver fibrosis (ELF) test					
All participants	33	9.0 (7.87–9.60)	27	8.51 (7.15–9.55)	
Patients with paired follow-up data	25	8.9 (7.87–9.60)	25	8.51 (7.15–9.55)	0.02

BMI: body mass index; WHtR: waist-to-height ratio; HOMA: homeostasis model assessment.

**Table 4:** Trend cross table and Pearson's chi-square test.

	Enhanced liver fibrosis (ELF) test				Transient elastography (Fibroscan)			
	Stable	Improved	Deteriorated	*n*	Stable	Improved	Deteriorated	*n*
BMI percentile								
Stable	0	2	0	2	0	0	0	0
Improved	0	***12***	4	16	1	***12***	1	14
Deteriorated	0	5	2	7	0	0	4	4
*n*	0	19	6	***25***	1	12	5	***18***
Pearson's chi-square test	*P * **= 0.001**				*P * **= 0.001**			
BMI-SDS								
Stable	0	0	0	0	0	0	0	0
Improved	0	***13***	4	17	1	***12***	1	14
Deteriorated	0	6	2	8	0	0	4	4
*n*	0	19	6	***25***	1	12	5	***18***
Pearson's chi-square test	*P * **= 0.001**			** **	*P * **= 0.001**			** **

BMI: body mass index; SDS: standard deviation score.

## Abbreviations

BMI: Body mass index
ELF: Enhanced liver fibrosis panel
NAFLD: Nonalcoholic fatty liver disease
NASH: Nonalcoholic steatohepatitis
TE: Transient elastography
HA: Hyaluronic acid
PIIINP: Amino-terminal propeptide of type III collagen
TIMP-1: Tissue metalloproteinase-1
WC: Waist circumference
HOMA: Homeostasis model assessment
WHtR: Waist-to-height ratio.
